# Knowledge fields and emerging trends about extracellular matrix in carotid artery disease from 1990 to 2021: analysis of the scientific literature

**DOI:** 10.1186/s40001-023-01259-4

**Published:** 2023-08-16

**Authors:** Ran Xu, Tianhua Li, Zhiqing Li, Wei Kong, Tao Wang, Xiao Zhang, Jichang Luo, Wenjing Li, Liqun Jiao

**Affiliations:** 1 Department of Neurosurgery, Xuanwu Hospital, Capital Medical University, Beijing, China; 2 China International Neuroscience Institute (China-INI), Beijing, China; 3 Department of Physiology and Pathophysiology, School of Basic Medical Sciences, Key Laboratory of Molecular Cardiovascular Science, Ministry of Education, Peking University Health Science Center, Beijing, China; 4 Laboratory of Computational Biology and Machine Intelligence, National Laboratory of Pattern Recognition, Institute of Automation, Chinese Academy of Sciences, Beijing, China; 5 Department of Interventional Radiology, Xuanwu Hospital, Capital Medical University, Beijing, China

**Keywords:** Carotid artery disease, Extracellular matrix, Stroke, Bibliometrics, Hotspots

## Abstract

**Background:**

Stroke is a heavy burden in modern society, and carotid artery disease is a major cause. The role of the extracellular matrix (ECM) in the development and progression of carotid artery disease has become a popular research focus. However, there is no published bibliometric analysis to derive the main publication features and trends in this scientific area. We aim to conduct a bibliometric analysis to reveal current status of ECM in carotid artery disease and to predict future hot spots.

**Methods:**

We searched and downloaded articles from the Web of Science Core Collection with “Carotid” and “Extracellular Matrix” as subject words from 1990 to 2021. The complete bibliographic data were analyzed by Bibliometrics, BICOMB, gCLUTO and CiteSpace softwares.

**Results:**

Since 1990, the United States has been the leader in the number of publications in the field of ECM in carotid artery disease, followed by China, Japan and Germany. Among institutions, Institut National De La Sante Et De La Recherche Medicale Inserm, University of Washington Seattle and Harvard University are in the top 3. “Arteriosclerosis Thrombosis and Vascular Biology” is the most popular journal and “Circulation” is the most cited journal. “Clowes AW”, “Hedin Ulf” and “Nilsson Jan” are the top three authors of published articles. Finally, we investigated the frontiers through the strongest citation bursts, conducted keyword biclustering analysis, and discovered five clusters of research hotspots. Our research provided a comprehensive analysis of the fundamental data, knowledge organization, and dynamic evolution of research about ECM in carotid artery disease.

**Conclusions:**

The field of ECM in carotid artery disease has received increasing attention. We summarized the history of the field and predicted five future hotspots through bibliometric analysis. This study provided a reference for researchers in this fields, and the methodology can be extended to other fields.

**Supplementary Information:**

The online version contains supplementary material available at 10.1186/s40001-023-01259-4.

## Background

Stroke is one of the main causes of death and morbidity in our aging society, among which ischemic stroke accounts for 87% of all strokes [1]. 13% to 32% of ischemic stroke caused by sudden large vessel occlusion was associated with carotid artery disease [2, 3]. Atherosclerosis is the main pathological basis of carotid artery disease. The incidence of ischemic stroke increases with the severity of carotid artery stenosis caused by atherosclerosis [2]. In the past hundred years, the understanding of the pathogenesis under atherosclerosis has mainly based on the theory of hyperlipidemia [4]. Therefore, the current clinical prevention and treatment of atherosclerosis related stroke mainly focuses on lipid-lowering. However, a large number of patients had stroke recurrence even though their blood lipid control reaches the standard [5–7]. Therefore, the novel treatment of carotid artery disease, in addition to the existing lipid-lowering treatment, is urgently needed.

The formation of carotid artery disease is a chronic and long-term process. The microenvironment of artery wall plays important roles in the progress of the disease. ECM is the main component of vascular microenvironment, which is critical for the functional stability of blood vessels and closely related with the occurrence of ischemic stroke. About 300 proteins make up the matrisome, which is the center of the vascular ECM [8, 9], including elastic fibers, fibrillar collagen, etc. Sufficient flexibility and viscosity of the artery is needed for the transmission of blood pressure to maintain continuous perfusion of the nearby capillaries, and ECM was essential for this process [10]. Previous study shows ECM plays important roles in vascular remodeling and plays critical roles in improving risk prediction and diagnosis of carotid artery disease management [11, 12]. More importantly, ECM can participate in information exchange and behavioral regulation with vascular smooth muscle cells (VSMCs) and other cells, thus involve in the progression of carotid artery disease from an overall perspective [13, 14]. Therefore, more and more researchers are committed to study the regulation and potential value of ECM in carotid artery disease.

Along with the recognition of the roles of ECM in the development of carotid artery disease, many striking research emerged in the past decades. Therefore, exploring new techniques for automated data analysis to extract useful knowledge is an exciting task. Bibliometric analysis has gained popularity in recent years, which measures the contribution of a research field and makes detailed predictions about the research trends or hotspots in a certain subject [15–17]. However, bibliometrics on ECM in carotid artery disease are still lacking. In this study, we analyzed the relevant articles from 1990 to 2021 to determine the research status and progress of ECM in carotid artery disease. Additionally, we clarified the research hotspots for ECM in carotid artery disease using the co-word biclustering analysis.

## Materials and methods

### Search methodology and strategies

The database: Web of Science Core collection, Edition: (SCI-EXPANDED) -1990- present. The search term was set as (TS = (Carotid)) AND TS = (Extracellular Matrix) and obtained 1191 literatures. Then, we set the document type as “article” and finally acquired 1056 articles. The relevant literature was first appeared in 1990. Thus, the time was set from 1990–01–01 to 2021–12–31, and 1041 articles were finally obtained. The above search results were completed on July 5, 2022.

### Data processing and bibliometric analysis

Web of Science was used to retrieve the data that included full records and cited references. We used Bibliometrics (http://bibliometric.com/) and CiteSpace (version 6.61.R2, Drexel University, USA) for bibliometric analysis. In this analysis, we took publication factors including countries, organizations, journals, authors, and the H-index [18] into consideration. The investigation also included the 2021 edition of the Journal Citation Report (JCR) and impact factor (IF), which are crucial metrics for gauging the scientific worth of research [19]. Then, the number of published papers as well as international collaborations were analyzed. Finally, we gathered highly mentioned terms to predicted the research frontiers and emerging trends [20, 21].

### Biclustering analysis of co-word

BICOMB [22] was employed to analyze the keywords of the articles. To create a binary matrix, we used the source articles as columns and the high-frequency phrases as rows. Then, gCluto [23] (version1.0, University of Minnesota) was used to perform biclustering analysis on the binary matrix. The parameter settings were as follows, Cluster method: repeated bisection; Similarity function: Cosine; Criterion function: I2. The biclustering process was repeated with different cluster numbers until the best matrix and mountain visualization results were obtained [17]. These results illuminated the semantic connections between the key terms and the source texts.

## Results

### Basic characteristics of the publications

**Fig. 1:**
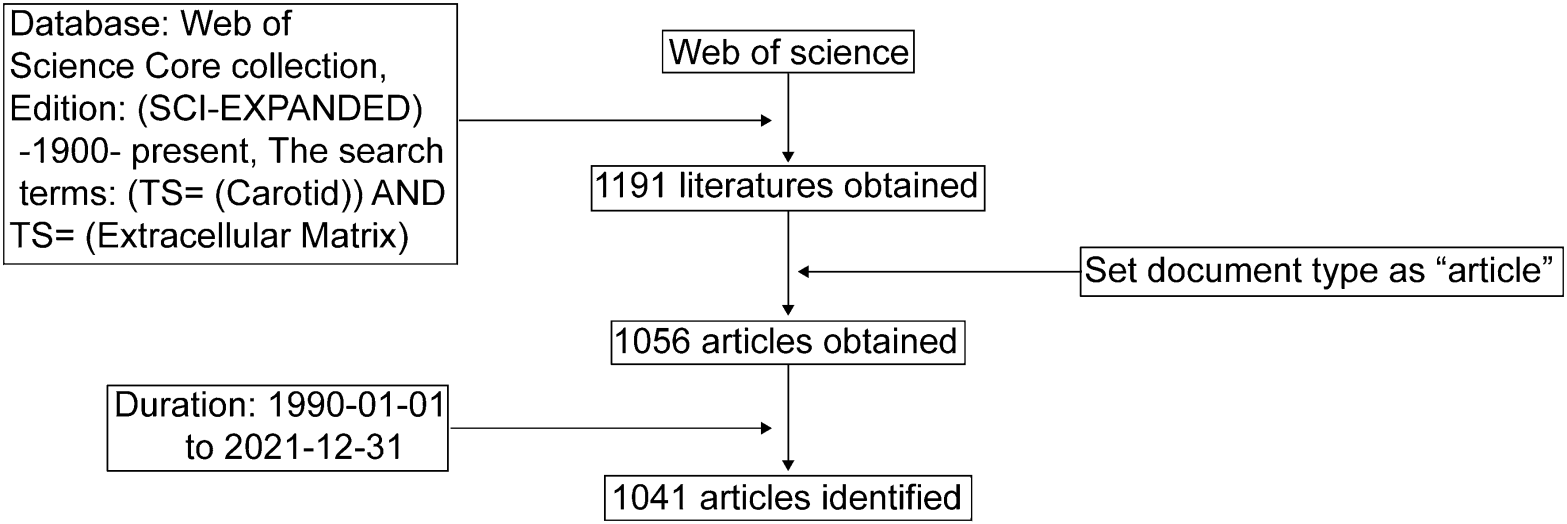
Flowchart of research strategy

**Fig. 2:**
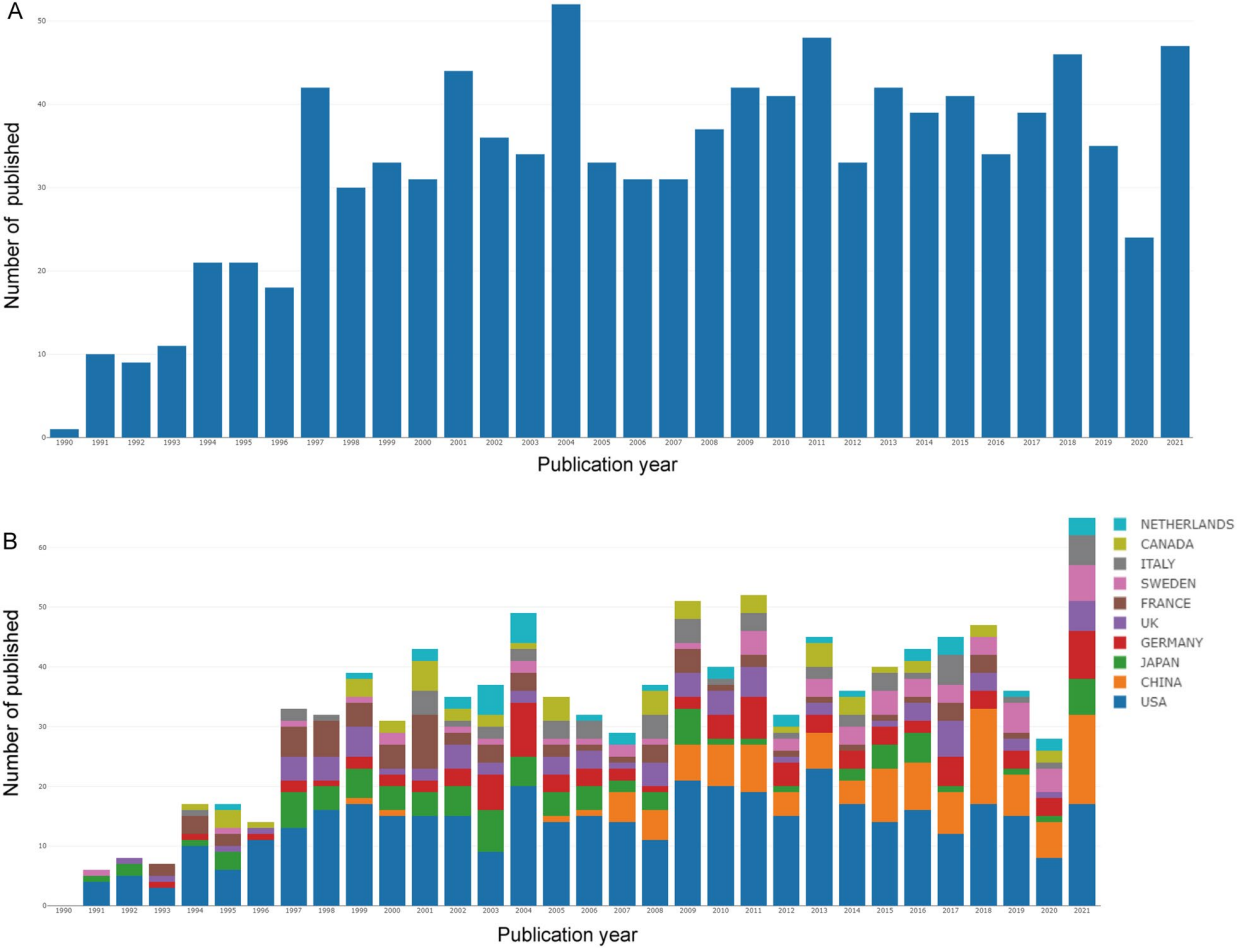
Output of related literature. **A** The number of annual publications and **B** growth trends of the top 10 countries/regions in field of ECM in carotid artery disease from 1990 to 2021. Conducted by Bibliometrics

Figure 1 depicted the flowchart of the search strategy. A total of 1041 papers were retrieved. The first article involving ECM in carotid artery disease was published in 1990, which described the effect of angiotensin II on VSMCs and the changes of ECM [24]. This paper established the relationship between ECM and VSMCs and promoted the development of the field. Subsequently, increasing number of excellent works about ECM in carotid artery disease have been published (Fig. 2A).

### Countries and institutions distribution bursts

**Fig. 3:**
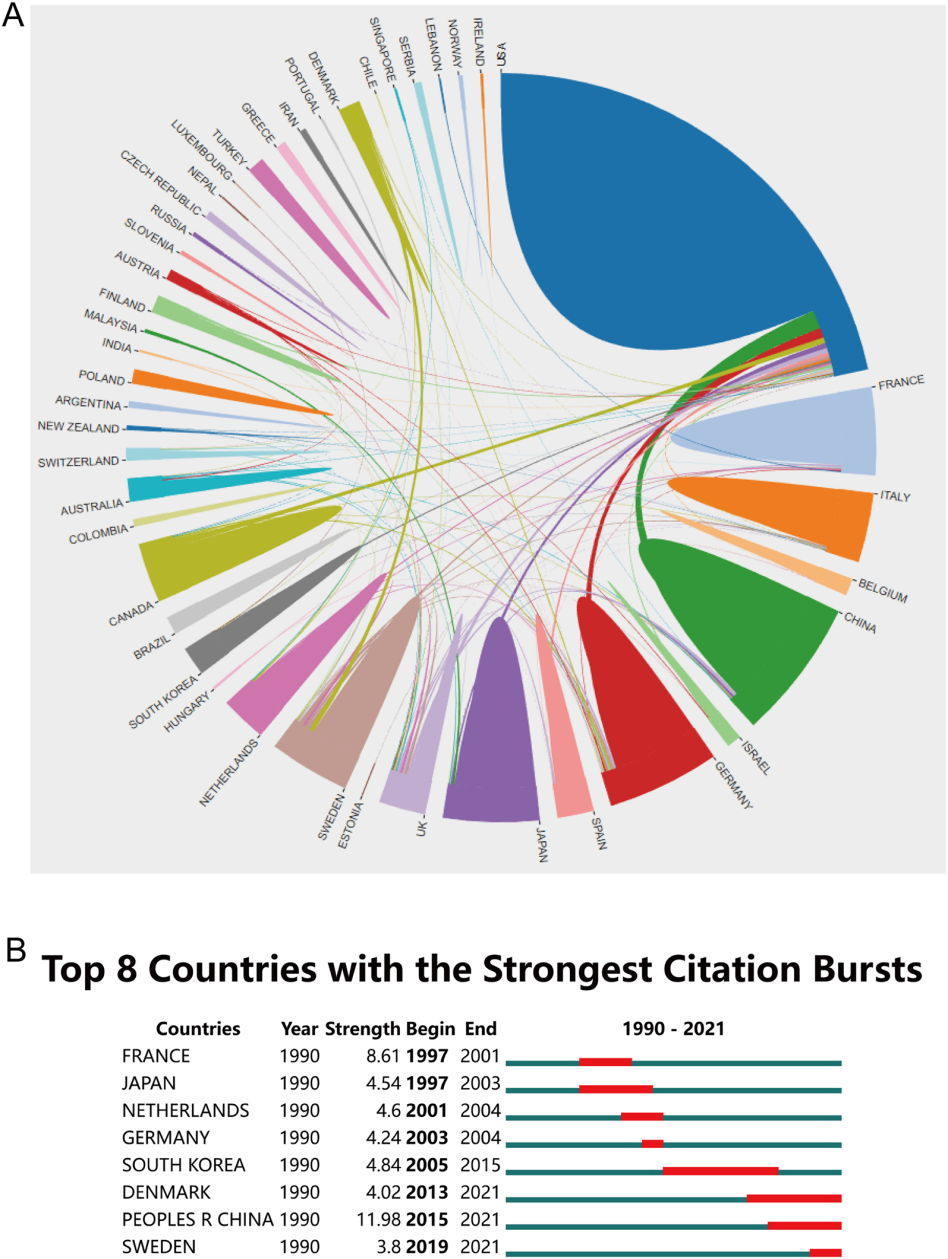
Distribution of publications from different countries/regions. **A** Cooperation between nations or regions. The link denotes a working relationship, whereas the area denotes the quantity of articles. Conducted by Bibliometrics. **B** The burstness detection of national publications by CiteSpace, “strength” represents the intensity of the burst, "begin" represents the beginning year of the burst, “end” indicates the ending year of the burst, red dotted line indicates the duration of the burst. The blue line indicates the entire period from 1990 to 2021

Forty-three countries and regions were involved in the field. The United States (436) published the largest number of articles, followed by China (108), Japan (90), Germany (89), etc. (Additional file 1). We observed that the United States has the highest H-index. Regarding the 10 countries with the highest number of published articles per year, we found that the United States consistently takes the top spot (Fig. 2B). In the analysis of cooperative relations between countries, we discovered that the US routinely coordinated with other countries (Fig. 3A). According to strongest citation bursts, we found that Sweden, China and Denmark are becoming frontier countries in this field (Fig. 3B).

**Fig. 4:**
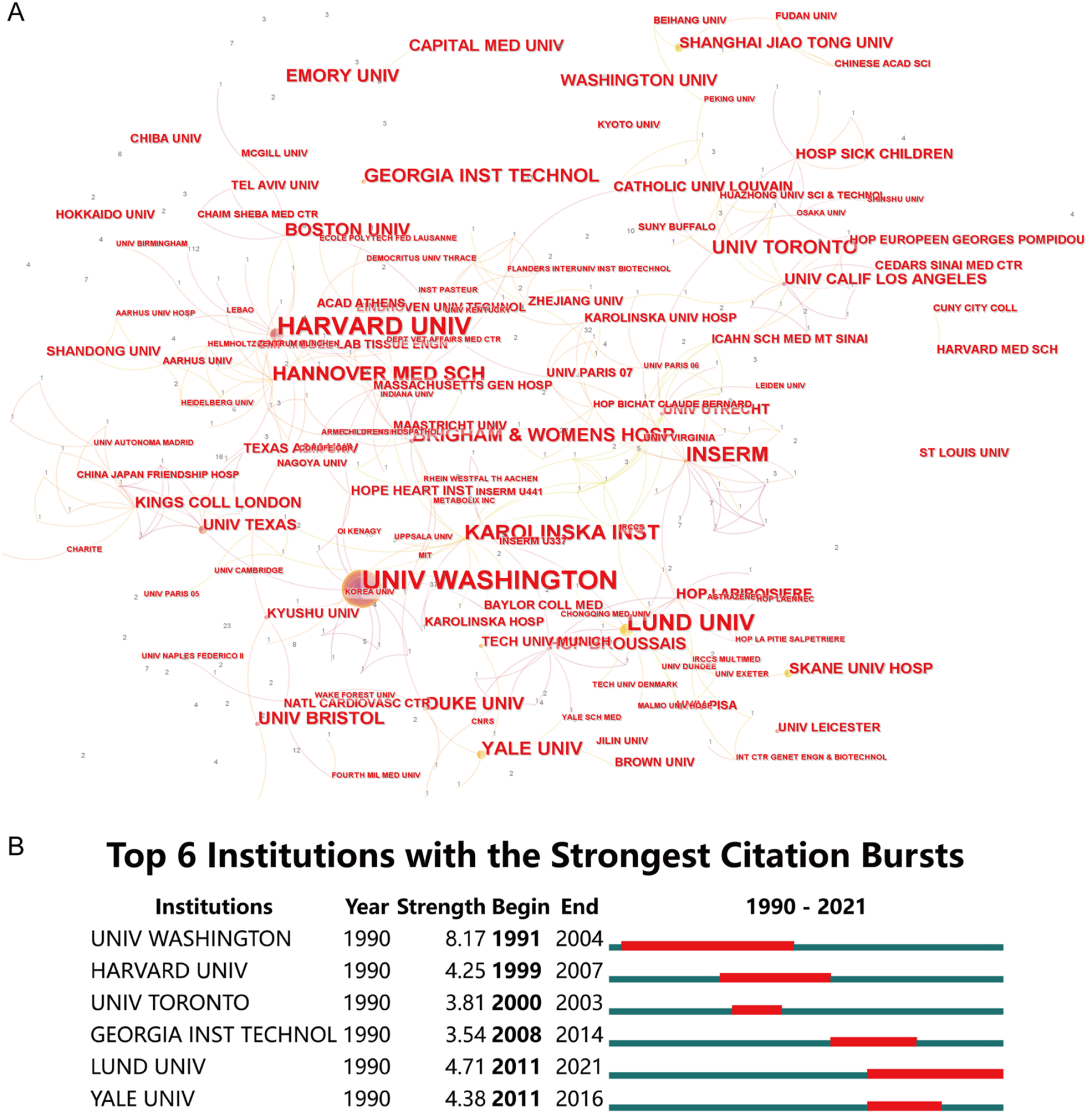
Distribution of publications from different institutions. **A** Cooperative relations among institutions. The line indicates that there are common articles issued between institutions. The value represents the number of co-articles. **B** The burstness detection about publications of institutions. Conducted by CiteSpace

When it comes to institutions, the top 10 for publications are listed in Additional File 2. Institut National De La Sante Et De La Recherche Medicale Inserm (53), University of Washington Seattle (51) and Harvard University (41), are in the top 3. From the perspective of interagency cooperation, the University of Washington and Harvard University have the most extensive cooperation with other institutions (Fig. 4A). According to strongest citation bursts, Yale University and London University are at the forefront of this research field (Fig. 4B).

### Journal distribution

382 journals were identified in field of ECM in carotid artery disease. The output of the top 10 (Additional file 3) most active related journals produced 327 articles, which accounts for 31.41% of the 1041 articles retrieved. The top 3 publications in terms of quantity are Arteriosclerosis Thrombosis and Vascular Biology (72), Atherosclerosis (43) and Circulation Research (38). The top 3 journals with the most average citations are Circulation (108.76), Circulation Research (99.03), Arteriosclerosis Thrombosis and Vascular Biology (66.35). The journal with the highest IF was Circulation (IF: 39.918), followed by Circulation Research (IF: 23.213) and Cardiovascular Research (IF: 13.081).

### Author distribution

**Fig. 5:**
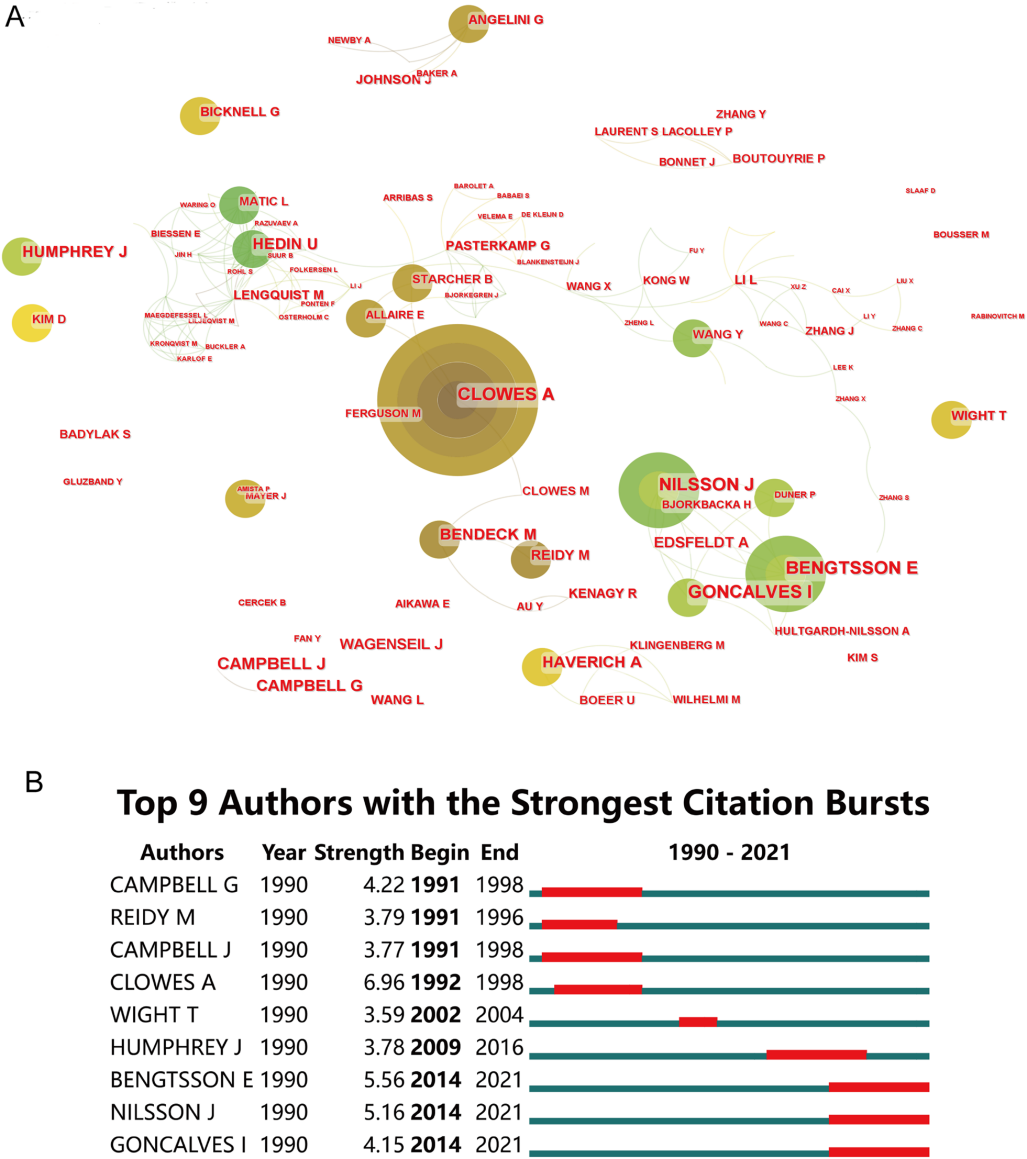
Distribution of publications from different authors. **A** The network map of productive authors. The circular area represents the number of articles issued. The darker the color is, the more significant the importance is. The lines indicate that the authors have jointly issued articles. **B** The burstness detection about publications of institutions. Conducted by CiteSpace

A total of 5638 authors were involved. The top 10 authors were ranked according to the quantity of publications (Additional file 4). The top 3 are Clowes, AW (22), Hedin, Ulf (18), Nilsson, Jan (18). The top 3 authors with the most citations on average are Reidy, MA (191.08), Clowes, AW (98.64), Wight, TN (64), and we found that all three authors are from the University of Washington. Through the analysis of the author's cooperative relationship, we found that the relationship network with CLOWES, AW as the core is the most extensive (Fig. 5A). According to strongest citation bursts, Goncalves Isabelare, Nilsson Jan and Bengtsson Eva were strongly quoted from 2014 to 2021 and were at the forefront of this research field (Fig. 5B).

### Analysis of the top 10 high-cited article

By analyzing the top 10 high-cited articles (Additional file 5), we found that “Experimental investigation of collagen waviness and orientation in the arterial adventitia using confocal laser scanning microscopy” has the highest citation (586). Two articles from Reidy MA ranked second (530) and third (502). In addition, we found that only one of the top 10 highly cited articles was published in the latest 10 years.

### Research hotspots of ECM in carotid artery disease

**Fig. 6:**
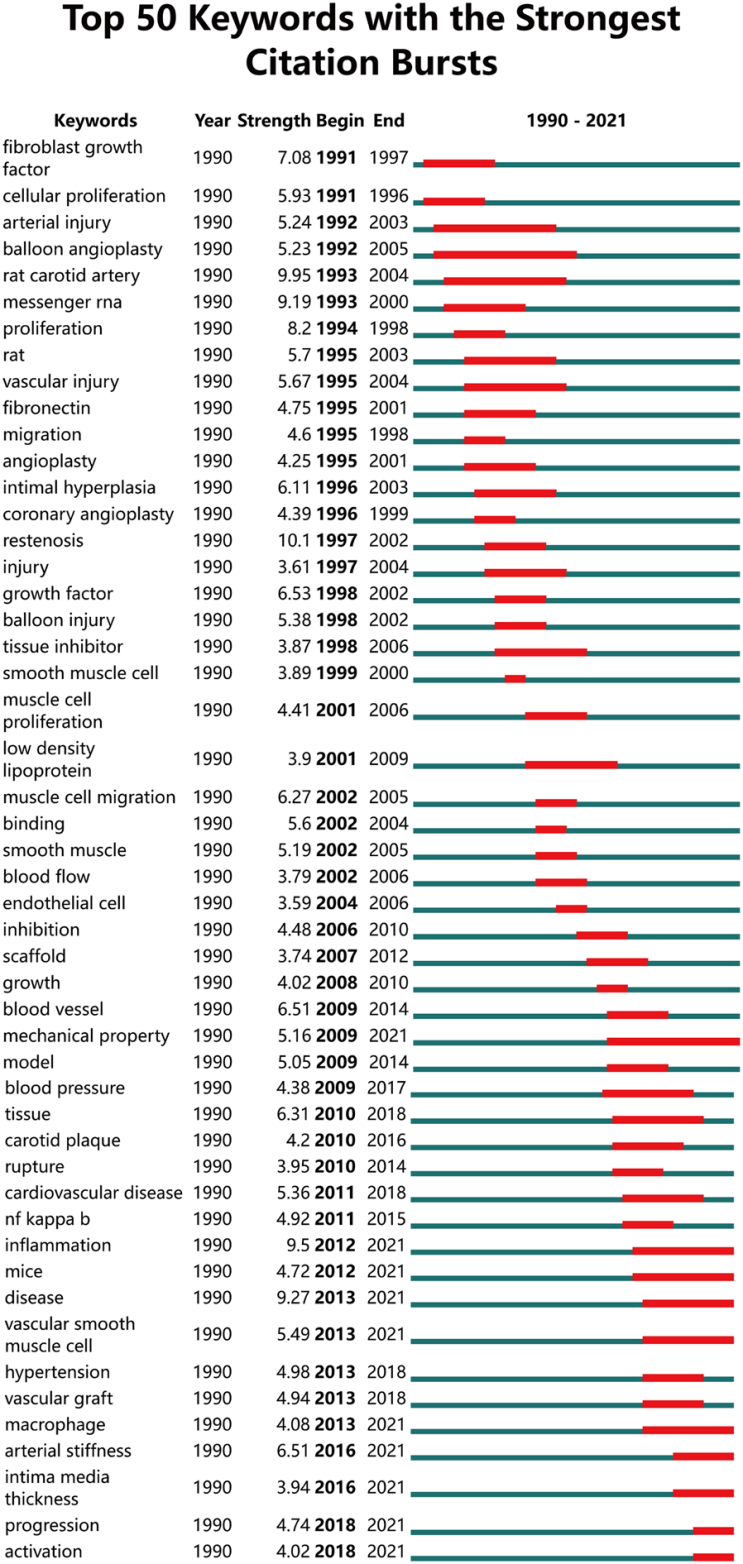
The top 50 burst of keywords analyzed by CiteSpace

**Fig. 7:**
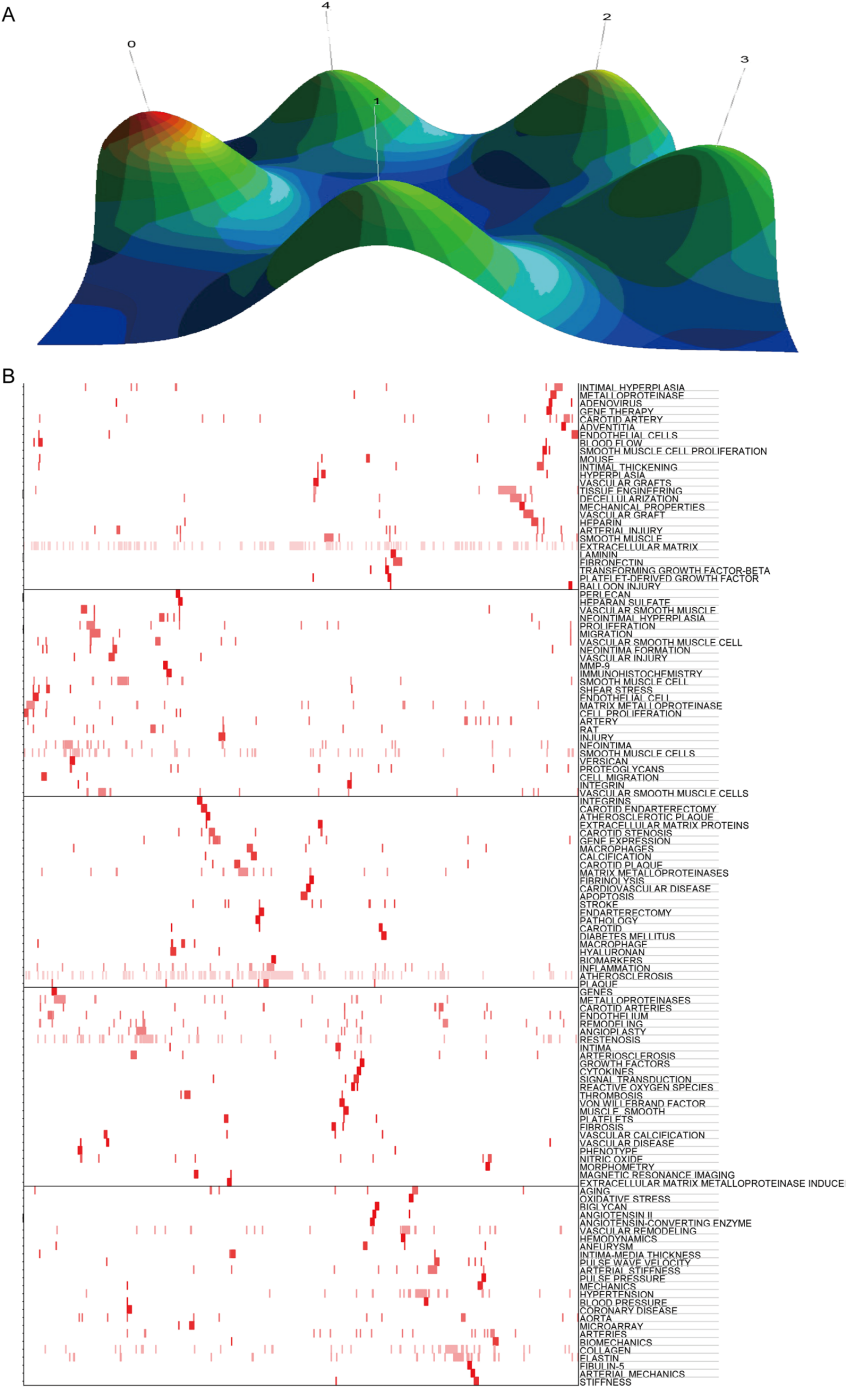
Biclustering of highly frequent major keywords and articles on ECM in carotid artery disease. Conducted by gCLUTO. **A** Mountain visualization. **B** Matrix visualization

The retrieved articles provided 1899 keywords in total. The keywords appeared more than five times were defined as extremely frequent keywords, and the cumulative percentage was 44.30%. For the top 50 terms from 1990 to 2021 with the strongest citation (Fig. 6), we noticed “mechanical property”, “inflammation”, “vascular smooth muscle cell” etc. are at the forefront in recent years. Through "gCLUTO," we conducted biclustering and five different clusters were sorted. In addition, we mapped the relationship between source articles and keywords using the mountain and matrix visualization (Fig. 7). Based on the above results, we examined the typical articles in each cluster and listed the following subjects as summaries for each cluster:

- Cluster 0 relates to the ECM in carotid arterial stiffness;
- Cluster 1 relates to the ECM in different stages of atherosclerosis;
- Cluster 2 relates to the ECM in the stability of carotid atherosclerotic plaque;
- Cluster 3 relates to the correlation between ECM of carotid plaque and age;
- Cluster 4 relates to how ECM affects the mechanical properties of carotid artery.

## Discussion

The mechanism research related to carotid artery disease has developed rapidly in recent decades, which provided novel approaches to improve the treatment of this disease. In the last 30 years, scholars have realized that the ECM microenvironment plays important roles in carotid artery disease [13]. With the in-depth study of ECM in carotid artery disease, the amount of relevant scientific literatures has increased dramatically, making it difficult to keep up with the latest research in real time. We summarized the characteristics of articles published in this field from 1990 to 2021 through statistical and quantitative analysis and identified five clusters of hot spots.

Cluster 0 relates to the ECM in carotid arterial stiffness. According to earlier research, carotid artery stiffness raises the chance of developing cerebrovascular disease and unintentional stroke [25]. Previous studies have shown that the metabolism of collagen, a component of the ECM, plays important roles in the pathogenesis of arterial remodeling and stiffness in hypertension. Collagenases or matrix metalloproteinases (MMPs) are inducers of arterial remodeling [26, 27]. In addition, collagen deposition, enhanced MMPs production, and reduced elastin in stiffened arteries can be observed by microscopy [28]. The above research illustrated that ECM may play important roles in the development of carotid artery stiffness, but the mechanisms remains unclear.

Cluster 1 relates to the ECM in different stages of atherosclerosis. The “response to retention theory” states that pathological intimal thickening (PIT) is the first stage of atherosclerosis [29, 30]. The ECM has central roles in initiation of atherosclerosis, primarily through the interaction between apolipoproteins and side chains of proteoglycans [31]. Plasma-derived lipoproteins are retained as a result of this interaction [32, 33]. In advanced stages of atherosclerosis, PITs can transform into fibroatheromas, which are recognized by the presence of a fibrous cap and a necrotic core. The fibrous cap is a highly cellular region enriched with VSMCs-derived αSMA+ cells, and advanced atherosclerosis exhibits increased levels of collagen and decreased levels of proteoglycans in the ECM component [34–37]. The above description suggests that the mechanisms of ECM in different stages of atherosclerosis need to be further explored.

Cluster 2 relates to the ECM in stability of carotid atherosclerotic plaque. Atherosclerotic plaques are composed of lipids, ECM and several cell types, mainly including bone marrow-derived cells, VSMCs and endothelial cells [38]. Rupture of vulnerable carotid atherosclerotic plaques is an important cause of ischemic stroke. It has been shown that plaque size is not representative of plaque stability, while the thickness of the fibrous cap, the lipid content of the necrotic core, the composition of the ECM and the presence of calcification seem to be more indicative of plaque vulnerability [39]. In addition, VSMCs accumulate at the rupture site of atherosclerotic plaques and release ECM proteins that confer intensity to restore plaque surface integrity [38]. The role of ECM-related proteins in atherosclerotic plaque stability still needs to be clarified, and in-depth work may explain the mechanisms in a new dimension.

Cluster 3 relates to correlation between ECM of carotid plaque and age. Age has been shown to be a major determinant for cross-sectional area of carotid artery [40], and carotid artery are known to get stiffer with increasing age [41]. According to previous studies, total and intermediate elastin decrease, and the collagen/elastin ratio increases with age [42, 43]. In clinical practice for carotid artery disease, doctors may consider different treatments and degrees for different age groups. However, studies on the pathological mechanism of atherosclerosis in different age groups are limited. Therefore, further exploration for internal relationship between ECM of carotid plaque and age will provide novel evidence to develop individualized therapy.

Cluster 4 relates to ECM affects the mechanical properties of carotid artery. Carotid artery curvature is usually associated with aging, hypertension, atherosclerosis and degenerative vascular diseases, while the mechanism is still unclear [44–46]. Previous studies showed that the degradation of elastin in ECM weakens the arterial wall, resulting in mechanical instability, which led to vascular curvature [47]. In this line, ECM may be a breakthrough to improve this situation. Furthermore, due to the rapid development of regenerative medicine technology and 3D printing, the manufacture of tissue engineered vascular grafts can be integrated, reshaped and repaired in vivo, which may lead to a paradigm shift of cardiovascular disease management [48]. Acellular scaffold, acellular blood vessels may become promising tissue engineering products for the treatment of carotid artery disease [49, 50]. Moreover, the alteration of ECM could play a role in the change of mechanical properties of cell-free scaffolds, cell-free vessels, affecting their mechanical strength and structure [51, 52]. Therefore, it is important to investigate the role of ECM in the mechanical alterations of carotid arteries for the treatment of carotid artery disease.

With the in-depth study of ECM, its importance in carotid artery disease has been further highlighted. Using ECM proteomics, Manuel et al. compared the ECM profiles of symptomatic and asymptomatic carotid plaques and identified four proteins, matrix metalloproteinase 9, S100 calcium binding protein A8/A9, cathepsin D, and galectin-3-binding protein. The above four proteins improved risk prediction and diagnosis in carotid artery disease management [12]. Meanwhile, the underlying mechanisms of ECM-regulated development and progression of carotid artery disease was further revealed. For example, Nidogen-2 of ECM maintains the contractile phenotype of VSMCs in vitro and in vivo through Jagged1-Notch3 signaling [53]. Thus, the above studies provided evidence to investigate whether Nidogen-2 has the potential to serve as a marker and therapeutic target for carotid atherosclerotic stenosis. COMP (cartilage oligomeric matrix protein), the substrate of ADAMS-7 (a disintegrin and metalloproteinase with thrombospondin type 1 motif 7), has been shown to play a protective role in vascular disease [54, 55]. Intriguingly, recent study demonstrated that a vaccine for ADAMS-7 (ATS7vac) was a novel atherosclerosis vaccine that also alleviates in-stent restenosis. The application of ATS7vac might be served as a complementary therapeutic strategy for current lipid-lowering strategy for carotid artery disease [56]. Besides, the effect of ECM on some tissue engineering products may help to improve innovation of future surgical materials and techniques for carotid artery disease [51, 52].

In interpreting the results of this study, several limitations should be noted. The databases are regularly updated, and we only included articles that were published between 1990 and 2021. Consequently, there may be a mismatch between our bibliometric analysis and actual publication circumstances. Future regular updates of the study to clarify the line of research on ECM in carotid artery disease are still needed.

## Conclusions

Through bibliometric analysis, we performed a comprehensive analysis of the knowledge organization of the underlying data for articles about ECM in carotid artery disease between 1990 and 2021. The past of the field is summarized, and future hotspots are predicted. These results can provide a useful reference for researchers in the field.

### Supplementary Information

**Additional file 1.** The top 10 countries/regions contributing to publications about ECM in carotid artery disease.

**Additional file 2. **The top 10 institutions contributing to publications about ECM in carotid artery disease.

**Additional file 3. **The top 10 most active journals that published articles about ECM in carotid artery disease.

**Additional file 4. **The top 10 most productive authors contributed to publications about ECM in carotid artery disease.

**Additional file 5. **The top 10 high-cited articles about ECM in carotid artery disease during 1990 to 2021.

## Data Availability

The datasets supporting the conclusions of this article were retrieved from using the Web of Science database.

## Abbreviations

ECM: Extracellular matrix
VSMCs: Vascular smooth muscle cells
MMPs: Matrix metalloproteinases
PIT: Pathological intimal thickening
