# Supplementary material for: Knowledge fields and emerging trends about extracellular matrix in carotid artery disease from 1990 to 2021: analysis of the scientific literature
Source: Eur J Med Res. 2023 Aug 16;28:284. doi: 10.1186/s40001-023-01259-4 (PMC10428572; doi:10.1186/s40001-023-01259-4)
Supplement: Supplementary file 1 — Additional file 1. The top 10 countries/regions contributing to publications about ECM in carotid artery disease. [file 40001_2023_1259_MOESM1_ESM.docx]

| Additional file 1. The top 10 countries/regions contributing to publications about ECM in carotid artery disease. | | | | | | |
| --- | --- | --- | --- | --- | --- | --- |
| Rank | Countries | Article counts | Percentage | H-index | Total number of citations | Average number of citations |
| 1 | USA | 436 | 41.88% | 87 | 23617 | 54.17 |
| 2 | CHINA | 108 | 10.38% | 29 | 2835 | 26.25 |
| 3 | JAPAN | 90 | 8.65% | 34 | 3449 | 38.32 |
| 4 | GERMANY | 89 | 8.55% | 32 | 3717 | 41.76 |
| 5 | FRANCE | 74 | 7.11% | 39 | 4316 | 58.32 |
| 6 | ENGLAND | 72 | 6.92% | 35 | 4189 | 58.18 |
| 7 | SWEDEN | 56 | 5.38% | 24 | 2145 | 38.30 |
| 8 | ITALY | 52 | 5.00% | 21 | 1382 | 26.58 |
| 9 | CANADA | 49 | 4.71% | 28 | 2002 | 40.86 |
| 10 | AUSTRALIA | 38 | 3.65% | 21 | 1323 | 34.82 |
