# Supplementary material for: Knowledge fields and emerging trends about extracellular matrix in carotid artery disease from 1990 to 2021: analysis of the scientific literature
Source: Eur J Med Res. 2023 Aug 16;28:284. doi: 10.1186/s40001-023-01259-4 (PMC10428572; doi:10.1186/s40001-023-01259-4)
Supplement: Supplementary file 2 — Additional file 2. The top 10 institutions contributing to publications about ECM in carotid artery disease. [file 40001_2023_1259_MOESM2_ESM.docx]

| Additional file 2. The top 10 institutions contributing to publications about ECM in carotid artery disease | | | | | | |
| --- | --- | --- | --- | --- | --- | --- |
| Rank | Institutions | Article counts | Percentage | H-index | Total number of citations | Average number of citations |
| 1 | Institut National De La Sante Et De La Recherche Medicale Inserm | 53 | 5.09% | 32 | 3334 | 62.91 |
| 2 | University of Washington Seattle | 51 | 4.90% | 34 | 5157 | 101.12 |
| 3 | Harvard University | 41 | 3.94% | 26 | 2919 | 71.20 |
| 4 | Assistance Publique Hopitaux Paris Aphp | 38 | 3.65% | 25 | 2408 | 63.37 |
| 5 | Udice French Research Universities | 35 | 3.36% | 24 | 2364 | 67.54 |
| 6 | Universite De Paris | 32 | 3.07% | 23 | 1917 | 59.91 |
| 7 | Lund University | 29 | 2.79% | 13 | 490 | 16.90 |
| 8 | University of California System | 29 | 2.79% | 17 | 1411 | 48.66 |
| 9 | Karolinska Institutet | 26 | 2.50% | 18 | 1154 | 44.38 |
| 10 | University of Texas System | 26 | 2.50% | 17 | 1126 | 43.31 |
