# Supplementary material for: Knowledge fields and emerging trends about extracellular matrix in carotid artery disease from 1990 to 2021: analysis of the scientific literature
Source: Eur J Med Res. 2023 Aug 16;28:284. doi: 10.1186/s40001-023-01259-4 (PMC10428572; doi:10.1186/s40001-023-01259-4)
Supplement: Supplementary file 3 — Additional file 3. The top 10 most active journals that published articles about ECM in carotid artery disease. [file 40001_2023_1259_MOESM3_ESM.docx]

| Additional file 3. The top 10 most active journals that published articles about ECM in carotid artery disease | | | | | | | | |
| --- | --- | --- | --- | --- | --- | --- | --- | --- |
| Rank | Journal title | Article counts | Percentage | H-index | Total number of citations | Average number of citations | IF | JCR |
| 1 | Arteriosclerosis Thrombosis and Vascular Biology | 72 | 6.92% | 39 | 4777 | 66.35 | 10.514 | Q1 |
| 2 | Atherosclerosis | 43 | 4.13% | 23 | 1388 | 32.28 | 6.847 | Q2 |
| 3 | Circulation Research | 38 | 3.65% | 31 | 3763 | 99.03 | 23.213 | Q1 |
| 4 | Journal of Vascular Surgery | 36 | 3.46% | 20 | 1374 | 38.17 | 4.860 | Q2 |
| 5 | Circulation | 33 | 3.17% | 29 | 3589 | 108.76 | 39.918 | Q1 |
| 6 | American Journal of Physiology Heart and Circulatory Physiology | 30 | 2.88% | 19 | 995 | 33.17 | 5.125 | Q2 |
| 7 | Cardiovascular Research | 27 | 2.59% | 21 | 1125 | 41.67 | 13.081 | Q1 |
| 8 | Plos One | 18 | 1.73% | 11 | 310 | 17.22 | 3.752 | Q2 |
| 9 | Journal of Vascular Research | 16 | 1.54% | 13 | 465 | 29.06 | 2.045 | Q3 |
| 10 | Annals of Biomedical Engineering | 14 | 1.35% | 10 | 766 | 54.71 | 4.219 | Q1 |
