# Supplementary material for: Knowledge fields and emerging trends about extracellular matrix in carotid artery disease from 1990 to 2021: analysis of the scientific literature
Source: Eur J Med Res. 2023 Aug 16;28:284. doi: 10.1186/s40001-023-01259-4 (PMC10428572; doi:10.1186/s40001-023-01259-4)
Supplement: Supplementary file 4 — Additional file 4. The top 10 most productive authors contributed to publications about ECM in carotid artery disease. [file 40001_2023_1259_MOESM4_ESM.docx]

| Additional file 4. The top 10 most productive authors contributed to publications about ECM in carotid artery disease | | | | | | |
| --- | --- | --- | --- | --- | --- | --- |
| Rank | Author | Institution | Article counts | H-index | Total number of citations | Average number of citations |
| 1 | Clowes, AW | Univ Washington,Dept Surg R25,seattle,WA 98195, USA | 22 | 20 | 2170 | 98.64 |
| 2 | Hedin, Ulf | Karolinska Inst, Dept Mol Med & Surg, SE-17176 Stockholm, Sweden | 18 | 12 | 796 | 44.22 |
| 3 | Nilsson, Jan | Lund Univ, Clin Res Ctr, Expt Cardiovasc Res Unit, Malmo, Sweden | 18 | 8 | 260 | 14.44 |
| 4 | Goncalves, Isabel | Lund Univ, Clin Res Ctr, Expt Cardiovasc Res Unit, Malmo, Sweden | 17 | 7 | 242 | 14.24 |
| 5 | Humphrey, J. D. | Texas A&M Univ, Dept Biomed Engn, Zachry Engn Ctr 337, College Stn, TX 77843 USA | 17 | 15 | 935 | 55.00 |
| 6 | Bengtsson, Eva | Lund Univ, Clin Res Ctr, Expt Cardiovasc Res Unit, Malmo, Sweden | 14 | 6 | 154 | 11.00 |
| 7 | Wagenseil, Jessica E. | Univ Washington, Dept Mech Engn & Mat Sci, St Louis, MO 63130 USA | 14 | 11 | 504 | 36.00 |
| 8 | Reidy, MA | Univ Washington,Dept Pathol,Seattle,WA 98195 USA | 13 | 13 | 2484 | 191.08 |
| 9 | Wight, TN | Univ Washington, Dept Med, Box 356426, Seattle, WA 98195 USA | 13 | 13 | 832 | 64.00 |
| 10 | Campbell, JH | Univ Queensland, Dept Anat Sci, Vasc Biol Res Ctr, Brisbane, Qld 4072, Australia | 12 | 12 | 383 | 31.92 |
