# Supplementary material for: Knowledge fields and emerging trends about extracellular matrix in carotid artery disease from 1990 to 2021: analysis of the scientific literature
Source: Eur J Med Res. 2023 Aug 16;28:284. doi: 10.1186/s40001-023-01259-4 (PMC10428572; doi:10.1186/s40001-023-01259-4)
Supplement: Supplementary file 5 — Additional file 5. The top 10 high-cited articles about ECM in carotid artery disease during 1990 to 2021. [file 40001_2023_1259_MOESM5_ESM.docx]

| Additional file 5. The top 10 high-cited articles about ECM in carotid artery disease during 1990 to 2021 | | | | | | | |  |
| --- | --- | --- | --- | --- | --- | --- | --- | --- |
| Rank | Article Title | Journal | Authors | Publication year | Total citation | Average annual frequency of citations | IF | JCR |
| 1 | Experimental investigation of collagen waviness and orientation in the arterial adventitia using confocal laser scanning microscopy | Biomechanics and Modeling in Mechanobiology | Rezakhaniha, R; Agianniotis, A; (...); Stergiopulos, N | 2012 | 586 | 53.27 | 3.623 | Q3 |
| 2 | Production of transforming growth factor beta 1 during repair of arterial injury | Journal of Clinical Investigation | MAJESKY, MW; LINDNER, V; (...); REIDY, MA | 1991 | 530 | 16.56 | 19.456 | Q1 |
| 3 | Role of basic fibroblast growth factor in vascular lesion formation | Circulation Research | LINDNER, V; LAPPI, DA; (...); REIDY, MA | 1991 | 502 | 15.69 | 23.213 | Q1 |
| 4 | Plasmin and matrix metalloproteinases in vascular remodeling | Thrombosis and Haemostasis | Lijnen, HR | 2001 | 368 | 16.73 | 6.681 | Q1 |
| 5 | Dialysis Accelerates Medial Vascular Calcification in Part by Triggering Smooth Muscle Cell Apoptosis | Circulation | Shroff, RC; McNair, R; (...); Shanahan, CM | 2008 | 367 | 24.47 | 39.918 | Q1 |
| 6 | Central role of RAGE-dependent neointimal expansion in arterial restenosis | Journal of Clinical Investigation | Sakaguchi, T; Yan, SF; (...); Naka, Y | 2003 | 312 | 15.60 | 19.456 | Q1 |
| 7 | Antibodies against transforming growth factor-beta 1 suppress intimal hyperplasia in a rat model | Journal of Clinical Investigation | WOLF, YG; RASMUSSEN, LM and RUOSLAHTI, E | 1994 | 280 | 9.66 | 19.456 | Q1 |
| 8 | Human connective tissue growth factor is expressed in advanced atherosclerotic lesions | Circulation | Oemar, BS; Werner, A; (...); Luscher, TF | 1997 | 277 | 10.65 | 39.918 | Q1 |
| 9 | Strong induction of members of the chitinase family of proteins in atherosclerosis - Chitotriosidase and human cartilage gp-39 expressed in lesion macrophages | Arteriosclerosis Thrombosis and Vascular Biology | Boot, RG; van Achterberg, TAE; (...); de Vries, CJM | 1999 | 276 | 11.50 | 10.514 | Q1 |
| 10 | Migration of cultured vascular smooth muscle cells through a basement membrane barrier requires type IV collagenase activity and is inhibited by cellular differentiation | Circulation Research | PAULY, RR; PASSANITI, A; (...); CROW, MT | 1994 | 275 | 9.48 | 23.213 | Q1 |
